# Idiopathic Facial Vein Aneurysm: A Case Report and Review of the Literature

**DOI:** 10.7759/cureus.33842

**Published:** 2023-01-16

**Authors:** Deema Martini, Mohammad K Shukairy, David Pasquale, Andrea Ziegler

**Affiliations:** 1 Otolaryngology - Head and Neck Surgery, Loyola University Medical Center, Maywood, USA; 2 Interventional Neuroradiology, Loyola University Medical Center, Maywood, USA

**Keywords:** vein thrombosis, valsalva maneuver, venous ectasia, neck mass, facial vein aneurysm

## Abstract

Venous aneurysms of the head and neck, and in particular the facial vein, are rare lesions that present as soft, compressible masses. We describe a case of an idiopathic aneurysm of the facial vein. A comprehensive literature review of this entity was conducted to better understand the disease process.

A 51-year-old female presented with a one-year history of a prominent but painless right submandibular mass that was more noticeable with maneuvers such as straining. An exam showed a painless, soft, compressible, right level 1b neck mass that enlarged with Valsalva. A computed tomography scan of the neck revealed a 2.7 cm enhancing mass adjacent to the right submandibular gland. Further workup included a direct puncture venography, which demonstrated an unruptured 3 cm venous aneurysm involving the right facial vein. Surgical excision was offered to the patient, which was completed without complications. Histopathology of the mass was consistent with a facial vein aneurysm and confirmed the suspected diagnosis. The patient was seen postoperatively in follow-up and was healing well.

While facial vein aneurysms are rare, it is important to recognize, work up, and treat them appropriately. A painless compressible mass that enlarges with Valsalva is suggestive of the diagnosis. Management may vary on a case-by-case basis with surgical resection being the definitive treatment of choice.

## Introduction

Venous aneurysms are rare and represent a focal area of dilation involving all three layers of the vascular wall. These lesions may occur anywhere in the body and are most commonly found on the extremities or intra-abdominally [1,2]. Less often, they can present in the head and neck, typically originating from the internal jugular vein and rarely from the facial vein [1]. Historically, these lesions often were mistaken for soft tissue masses [1]. Accurate diagnosis is imperative as neck masses may have a broad differential diagnosis including processes that are infectious, congenital, traumatic, benign, or malignant [3]. In particular, the increasing incidence of human papillomavirus-related oropharyngeal cancers, and their tendency to present as neck masses, must be considered [4]. With the advent of noninvasive imaging modalities, venous aneurysms can now be identified more readily. In this report, we describe the case of a 51-year-old female with a right facial vein aneurysm presenting as a submandibular mass.

## Case presentation

Presentation and workup

**Figure 1:**
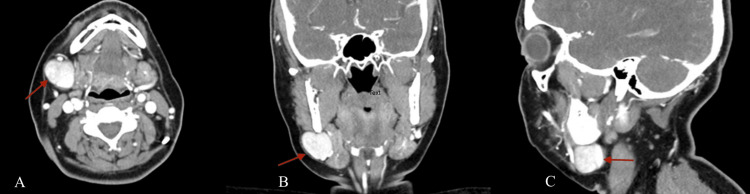
Computed tomography (CT) scan with IV contrast showing a hyper-enhancing mass originating from the right facial vein (red arrows) A: Axial, B: Coronal, C. Sagittal

**Figure 2:**
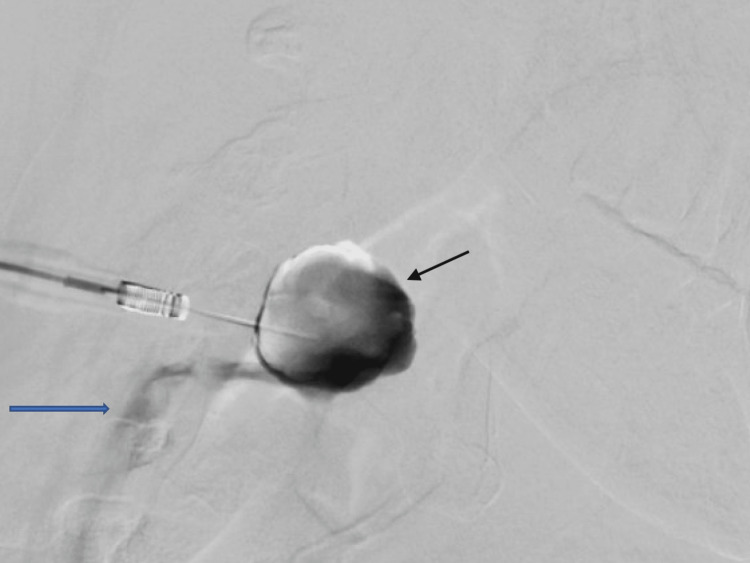
Direct puncture venography highlighting the facial vein aneurysm (black arrow) and immediate opacification of the right internal jugular vein (blue arrow)

A 51-year-old female presented with a one-year history of a prominent right submandibular mass that was more noticeable with Valsalva maneuvers such as straining. The mass was asymptomatic and painless. She denied any episodes of bleeding or discoloration and there was no history of trauma or inflammation in the region. She had a past medical history of well-controlled hypothyroidism and denied any alcohol or tobacco use. The physical exam showed a painless, soft, compressible, right level 1b neck mass that enlarged with Valsalva, adjacent to the submandibular gland. A computed tomography (CT) scan of the neck revealed a 2.7 cm well-circumscribed enhancing mass separate from the more medially located submandibular gland (Figure 1). This was suspicious for a slow-flow vascular malformation. The patient was referred to Neuro Interventional Radiology for a formal evaluation and consideration for sclerotherapy if appropriate. The patient ultimately underwent a direct puncture venography, which demonstrated an unruptured 3 cm venous aneurysm involving the right facial vein approximately 1.5 cm proximal to its connection into the right internal jugular vein (Figure 2). Given these findings, the decision was made to abort any sclerotherapy and the patient was offered surgical excision.

Surgical approach

**Figure 3:**
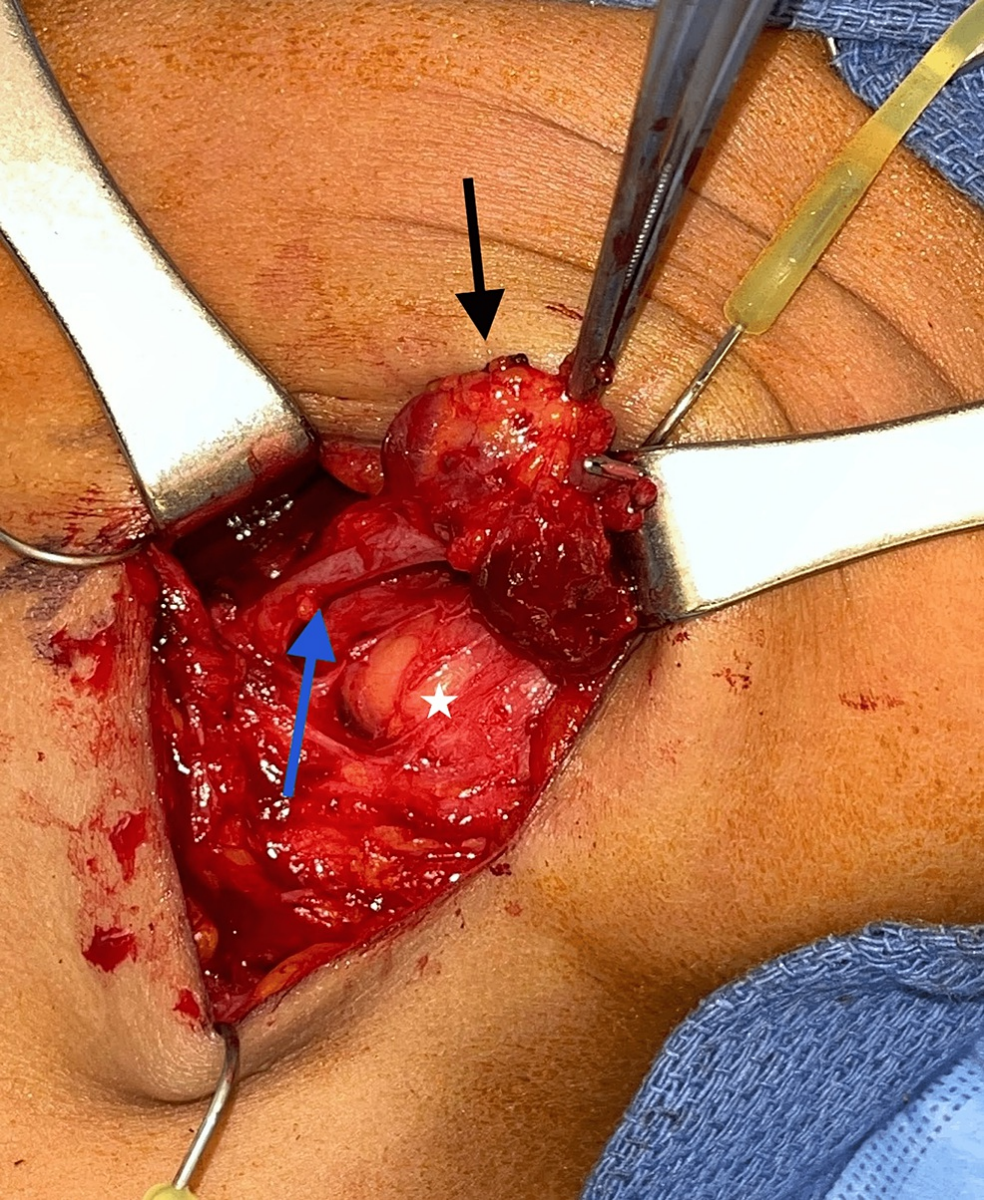
Transcervical exposure showing the aneurysmal mass stemming off the right facial vein Image shows the aneurysm (black arrow) overlying the facial vein (blue arrow). Note the close proximity of the submandibular gland (white star).

The patient underwent transcervical excision of the right venous aneurysm. The lesion was identified just deep to the platysma and noted to be arising from the facial vein. The facial vein, along with multiple smaller contributing vessels, was dissected and carefully isolated from surrounding structures and subsequently ligated (Figure 3). Histopathology of the mass confirmed the diagnosis and showed a dilated vessel wall with an organizing thrombus consistent with a facial vein aneurysm.

Postoperative course

The patient’s postoperative course was uncomplicated, and she was discharged on the same day. She was seen at her four-week follow-up appointment and was feeling well at that time, without complications. The scar was healing as expected.

## Discussion

Venous aneurysms in the head and neck are uncommon, and involvement of the facial vein is exceedingly rare, with only a few cases described in the literature (Table 1) [2,5-10]. They are typically benign and may present with transient enlargement during movements that increase intrathoracic pressure or impede venous return. Venous aneurysms are typically idiopathic but may be secondary to surgery, trauma, infection, congenital vascular abnormalities, or other underlying inflammatory processes [5-11]. In the case of facial vein aneurysms, associations with chronic sialadenitis have been described in the literature [6]. Duplex ultrasound (US) is a non-invasive, readily available test that offers good reliability in the head and neck, making it the initial imaging study of choice when there is high suspicion for a venous aneurysm. Further confirmatory testing that can offer more informative detail for preoperative planning may include CT scans, magnetic resonance imaging, or invasive venography. In addition to clinical signs and symptoms, these diagnostic studies are especially important in ruling out more aggressive pathology that can present in a similar fashion [3,4]. The definitive treatment of choice for venous aneurysms is surgery, and the indication may vary case-by-case [12]. In contrast to venous aneurysms in other anatomical areas, such as the intra-abdominal region, those in the head and neck are not known to pose a high risk for venous thromboembolism or rupture, and the decision to pursue surgical intervention is often for cosmetic reasons or to address symptoms associated with an enlarging mass [1,13]. Therefore, continued observation with interval imaging may be an appropriate alternative.

**Table 1:** Summary of facial vein aneurysms to date

	Age (y)	Sex	Location	size	Symptoms	Treatment	Follow-up	Comments
Mehta et al. [2]	17	F	Right common facial vein, anterosuperiorly close to masticator space	36.2 x 30.4 mm	Smooth, painless, diffuse swelling; increased in size with Valsalva	Surgical excision	Uneventful	
Shekib et al. [5]	38	M	Proximal end of the left facial vein	N/A	Soft, nontender swelling while supine and with Valsalva	Surgical excision	N/A	
Ilica et al. [6]	20	M	Left submandibular space	N/A	Swelling and pain	Antibiotic therapy for sialadenitis	Resolution of submandibular sialadenitis but the persistence of facial vein aneurysm asymptomatically	Proposed etiology linking the inflammatory condition to adjacent weakening of the vessel wall with subsequent aneurysmal dilatation
Morton et al. [7]	62	F	Right submandibular space	18 x 14 mm	Painless mass	Surgical excision	Uneventful	
Kaushal et al. [8]	25	M	Retromandibular vein near the confluence with the common facial vein	50 x 40 mm	Diffuse painless, non-tender swelling, more noticeable with Valsalva	Surgical excision	2 months; Uneventful	
Poyton et al. [9]	31	M	Lateral and anterior to the angle of the mandible	N/A	Smooth, warm, slightly tender swelling; increase in size with lowering of the head	Pt refused surgical excision	N/A	
Daily et al. [10]	11	M	Right neck below the angle of the mandible	25 x 40 mm	Painless, compressible mass present at birth; increased in size with Valsalva (crying)	Surgical excision	N/A	Congenital finding with a progressive increase in size
Present Case	51	F	Right submandibular space	30 mm	Painless mass that increased in size with Valsalva	Surgical excision	4 weeks; Uneventful	

## Conclusions

While venous aneurysms in the head and neck region, particularly of the facial vein, are exceedingly rare, they are important to appropriately recognize and manage. A painless, compressible mass that enlarges with Valsalva is suggestive of the diagnosis, which can be confirmed with duplex ultrasound. Management of these lesions may vary on a case-by-case scenario based on patient preference and the potential morbidity of continued observation vs surgery. We shared a case of our patient with a right-sided facial vein aneurysm who underwent uncomplicated surgical resection.
